# A Prospective Clinical Evaluation of Combined Targeted Verifiable Subcision and Helium Plasma Radiofrequency for the Treatment of Cellulite and Skin Laxity

**DOI:** 10.1093/asjof/ojag047

**Published:** 2026-03-26

**Authors:** Barry E DiBernardo

## Abstract

Cellulite affects 85% to 98% of postpubertal females and is characterized by fibrous septa contraction, dermal thinning, and fat lobule herniation, resulting in visible skin dimpling and textural irregularities. Although multiple treatment modalities exist, most target only select aspects of this multifactorial condition. This study evaluates the safety and efficacy of a novel approach combining targeted verifiable subcision (TVS) and helium plasma radiofrequency (RF) to disrupt fibrous septa, stimulate collagen remodeling, and improve skin elasticity. A prospective, single-center, investigator-initiated trial enrolled 22 patients who received a single-session treatment. Efficacy was assessed using quantitative imaging, the Physician and Subject Global Aesthetic Improvement Scale (PGAIS, SGAIS) at Day (D) 30, D90, and D180. Histological analysis evaluated collagen remodeling, elastin regeneration, and epidermal restructuring. Safety assessments included adverse events and procedural tolerability. Quantitative imaging showed progressive improvement through D180. By D180, improvement was observed in 77.3% (right thigh) and 81.8% (left thigh). PGAIS scores increased from 38% at D30% to 68% at D90 and D180. SGAIS peaked at D30 (57%), declining to 55% at D90% and 45% at D180. Histology confirmed progressive remodeling and increased extracellular matrix integrity. Two mild, self-resolving adverse events were reported (9%).Combining TVS and helium plasma RF may be a safe, well-tolerated, and effective treatment for improving cellulite appearance and skin laxity. Objective, histological, and physician-assessed outcomes confirmed dermal regeneration and clinical improvement, supporting integration of mechanical and energy-based therapies for minimally invasive cellulite treatment.

**Level of Evidence**: 4 (Therapeutic) 
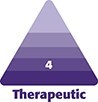

Cellulite affects 85% to 98% of postpubertal females across all racial backgrounds and is characterized by structural changes in connective tissue that lead to visible dimpling and skin laxity.^1^ Although not a pathological condition, cellulite develops because of adipose cell expansion and connective tissue alterations, primarily in the thighs and buttocks. This process leads to fibrous septa contraction and hypodermal fat herniation, resulting in the classic “cottage cheese” dimpling appearance.^2,3^

The proposed etiology of cellulite is multifactorial and includes (1) fibrous septa that become sclerotic then contract over time while tethering the dermis thereby forming depressions, (2) dermal thinning and reduced extracellular matrix integrity, leading to (3) hypodermal fat lobules that herniate upward, creating a puckered skin surface.^4,5^ The structural mechanisms underlying cellulite have been examined through histological analysis and MRI studies, which show septal tethering and upward fat protrusion into the dermis.^6^ Although harmless from a medical standpoint, cellulite remains a cosmetic concern and is frequently regarded as an undesirable aesthetic feature by those affected.

Advances in minimally invasive technologies have sought to address these contributing factors simultaneously through targeted subdermal energy delivery. In studies using 1440 nm pulsed Nd:YAG lasers, the authors have demonstrated that thermal subcision can both release fibrous septa and stimulate dermal remodeling.^7-9^ More recently, tissue-stabilized guided subcision and targeted verifiable subcision (TVS) systems have demonstrated long-term efficacy in treating cellulite by directly releasing fibrous septa under image or tactile guidance.^10,11^ These referenced studies highlight the multifactorial causality of cellulite, underscoring the rationale for multimodal therapeutic approaches if all etiologies are to be addressed.

This investigator-initiated trial was designed to evaluate the efficacy and safety of a novel combination treatment for cellulite and skin laxity, utilizing TVS (Avéli, Tiger Aesthetics Medical, Mountain View, CA) followed by helium plasma radiofrequency (RF; Renuvion, Apyx Medical, Clearwater, FL) application. This approach integrates 2 distinct mechanisms of action: (1) mechanically disrupting fibrous septa bands beneath the dermis to release tethered skin and (2) stimulating collagen production and enhancing skin elasticity through helium plasma RF. By targeting both the structural and textural components of cellulite, this combined therapy aims to provide a more effective and sustained solution compared with traditional monotherapies using contemporary technologies.

## METHODS

This was a prospective, single-center, investigator-initiated trial. The study was approved by the Western Copernicus Group IRB (Princeton, NJ) and was performed in accordance with the Food, Drug and Cosmetic Act and Good Clinical Practice guidelines (ICH E6-R2). Twenty-two patients were enrolled and treated, each receiving a single-session combination treatment. Patients were evaluated at baseline and followed up on Day (D) 7, D30, D90, and D180.

The treatment consisted of a single-session combination approach, utilizing TVS followed by helium plasma RF application. The mechanical release was performed using a controlled, standardized technique to target and disrupt fibrous septa, whereas helium plasma RF was employed to stimulate collagen remodeling and improve skin laxity. The helium plasma RF system operated at 80% power with a 1.5 L/min helium flow rate, delivering controlled energy to the subdermal tissue.

### Treatment Procedure

1. Pretreatment preparation
  - Patients were positioned in a supine or prone position, depending on the treatment area.
  - The targeted areas were mapped preoperatively, marking cellulite dimples and treatment zones.
  - Local anesthesia with lidocaine and epinephrine was used in all patients.
2. Targeted verifiable subcision
  - TVS was performed using the Avéli hand-held device to mechanically disrupt fibrous septa responsible for cellulite dimpling.
  - Each patient had 4 dimples treated per thigh, and 2 patients received additional treatment in the buttocks.
3. Helium plasma RF application
  - Following the septal release, helium plasma RF was applied to the thighs using the Apyx ONE generator and APYX-15-TP handpiece.
4. Posttreatment protocol
  - Immediately posttreatment, cold compresses were applied to minimize swelling.
  - Patients were advised to avoid strenuous activity for 48 h but could resume normal daily activities immediately.
  - Postprocedure pain management included oral analgesics if needed.

Standardized 2- and 3-dimensional images were taken utilizing the Canfield Primos Clinical Research system (Canfield Scientific, Parsippany, NJ). Outcome measures included quantitative imaging analysis at D30, D90, and D180 to measure the percentage of change observed in cellulite severity. Skin laxity was graded by the study investigator using the Nürnberger–Müller classification system. In this system, cellulite is graded as follows: Grade 0—no visible cellulite; Grade 1 (mild)—visible dimpling with skin pinching only; Grade 2 (moderate)—dimpling visible while standing but not lying down; and Grade 3 (severe)—dimpling visible in both standing and lying positions. Physician Global Aesthetic Improvement Scale (PGAIS; scored by the study investigator) and Subject Global Aesthetic Improvement Scale (SGAIS) were completed at D30, D90, and D180.

Histological analysis for 5 sample patients of collagen, elastin, and epidermal remodeling from baseline to D90 and D180. Tissue samples were collected from the TVS and helium plasma RF treatment area using 3 mm punch biopsies from consistent anatomical locations on the posterior thigh at D0, D90, and D180. All biopsies were performed by the same investigator to minimize variability. Samples were fixed in 10% buffered formalin, paraffin embedded, and sectioned at 4 µm thickness. Hematoxylin and eosin, Verhoeff–Van Gieson, and periodic acid–Schiff (PAS) staining were performed using standardized protocols across all samples. Photomicrographs were captured under identical lighting and magnification conditions. All slides were reviewed by an experienced histopathologist ensuring objective and consistent interpretation. A scoring system was used to assess collagen and elastin content, ranging from 0 to 3, where 0 = absent, 1 = minimal presence, 2 = moderate presence, and 3 = robust presence. Epidermal thickness was measured and compared across time points. Safety assessments, including adverse events were also collected.

All data were analyzed using descriptive statistics. Continuous variables such as age, BMI, and changes in quantitative imaging measurements were summarized using mean and standard deviation. Categorical variables, including PGAIS and SGAIS responses and adverse event frequencies, were summarized using counts and percentages.

## RESULTS

The mean age of the 22 female participants was 43.6 ± 7.6 years, ranging from 27 to 54 years. Patients had an average BMI of 25.3 ± 3.2 kg/m^2^, with a range of 19.7 to 31.9 kg/m^2^. The racial distribution of the study population included 59% Caucasian, 36% identifying as Other, and 5% Asian/Pacific Islander, with 68% of patients being non-Hispanic and 32% Hispanic (Table 1, Table 2).

**Table 1.** Subject Demographics

	Baseline
Age (yrs)	
N	22
Mean ± SD	43.6 ± 7.6
Median	45.5
Min, Max	(27, 54)
Sex	
Female	100% (22/22)
Male	0% (0/22)
Race	
N	22
Caucasian	59% (13/22)
Other	36% (8/22)
Asian/Pacific Islander	5% (1/22)
Ethnicity	
Non-Hispanic	68% (15/22)
Hispanic/Latin	32% (7/22)

**Table 2.** Subject BMI

	Baseline	D30	D90	D180
BMI				
N	22	21	22	22
Mean ± SD	25.3 ± 3.2	25.5 ± 2.6	24.7 ± 3.0	24.6 ± 3.1
Median	25.6	25.6	25.3	24.4
Min, Max	(19.7, 31.9)	(21.6, 30.7)	(18.9, 30.5)	(18.7, 31.0)

Baseline Nürnberger–Müller cellulite severity was assessed by physician evaluation of dimple severity in each thigh. In the right thigh, 7 patients had mild cellulite, 9 had moderate cellulite, and 6 had severe cellulite. In the left thigh, 7 patients were classified as mild, 11 as moderate, and 4 as severe. These baseline assessments provided an important reference for evaluating treatment response variability across different severity levels.

The helium plasma treatment involved multiple controlled passes (mean: 2.2 ± 0.5 right, 2.1 ± 0.5 left), with an average total energy delivery of 4.1 kJ (right thigh) and 3.7 kJ (left thigh).

Quantitative imaging analysis demonstrated progressive and sustained improvement in cellulite appearance and skin laxity from D30 to D180. At D30, 76.2% of patients in the right thigh and 90.5% in the left thigh showed some level of improvement. The outcome measure at D90 revealed that 63.6% of patients in the right thigh and 86.4% in the left thigh demonstrated improvement when compared with baseline images. By D180, improvement was observed in 77.3% of patients in the right thigh and 81.8% in the left thigh, indicating continued structural remodeling.

**Figure 1.**
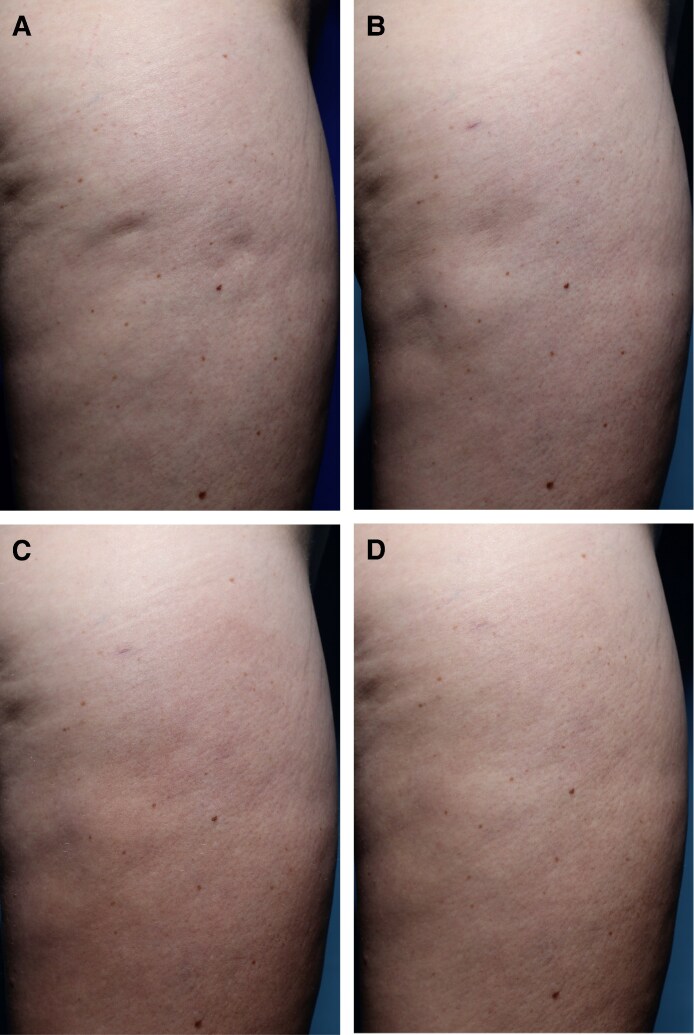
Example images of cellulite (A) at screening/baseline and following targeted verifiable subcision and helium plasma radiofrequency treatment (B) at Day 30, (C) Day 90, and (D) Day 180, demonstrating observed improvement. Patient: a 43-year-old, Caucasian.

**Figure 2.**
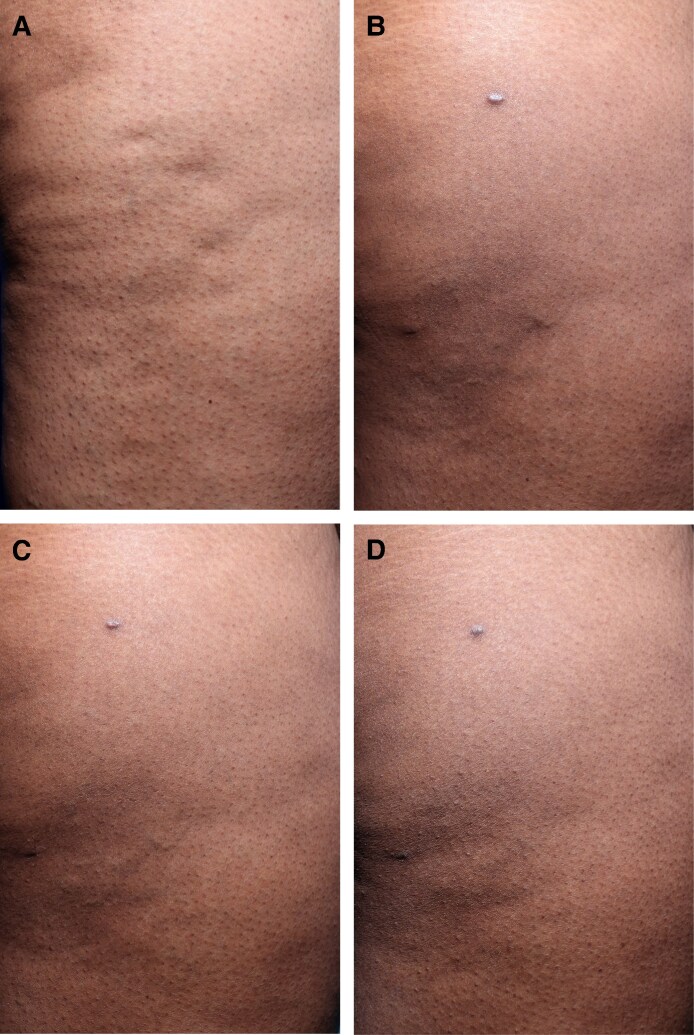
Example images of cellulite (A) at screening/baseline and following targeted verifiable subcision and helium plasma radiofrequency treatment (B) at Day 30, (C) Day 90, and (D) Day 180, demonstrating observed improvement. Patient: a 31-year-old, mixed race.

Physician-reported aesthetic improvements, assessed using the PGAIS, demonstrated a gradual and sustained increase over time, with the highest scores observed at D90 and D180. The percentage of patients demonstrating improvement increased from 38% at D30% to 68% at D90 and D180 (Figures 1A-D and 2A-D).

Patient-reported outcomes using the SGAIS peaked at D30, with 57% of patients reporting improvement, followed by a slight decline to 55% at D90 and 45% at D180.

Histological analysis demonstrated structural improvements in the dermal and extracellular matrix over time in the 5 patients who underwent biopsy. At baseline, collagen scores were low, with 3 patients scoring 1 and 2 patients scoring 2. At D90 (*n* = 4), collagen scores increased, with 2 patients scoring 2 and 2 patients scoring 3. By D180 (*n* = 4), further collagen maturation was observed, with 3 patients demonstrating a collagen score of 3 and 1 patient a score of 2.

**Figure 3.**
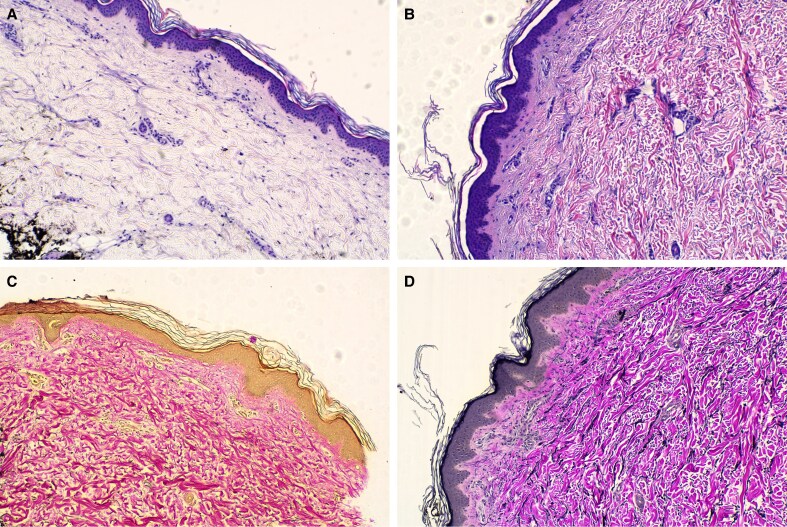
Example image of a collagen histology slide (A) at screening/baseline with a score of 1 and (B) following targeted verifiable subcision (TVS) and helium plasma radiofrequency (RF) treatment at Day 180 with a score of 3. Example image of an elastin histology slide (C) at screening/baseline with a score of 0 and following TVS and (D) helium plasma RF treatment at Day 90 with a score of 2. Patient: a 41-year-old, Caucasian. In the posttreatment slides (B, D), observe the denser, more organized collagen and elastin fibers compared with the baseline (A, C), indicating extracellular matrix remodeling and improved tissue structure.

**Figure 4.**
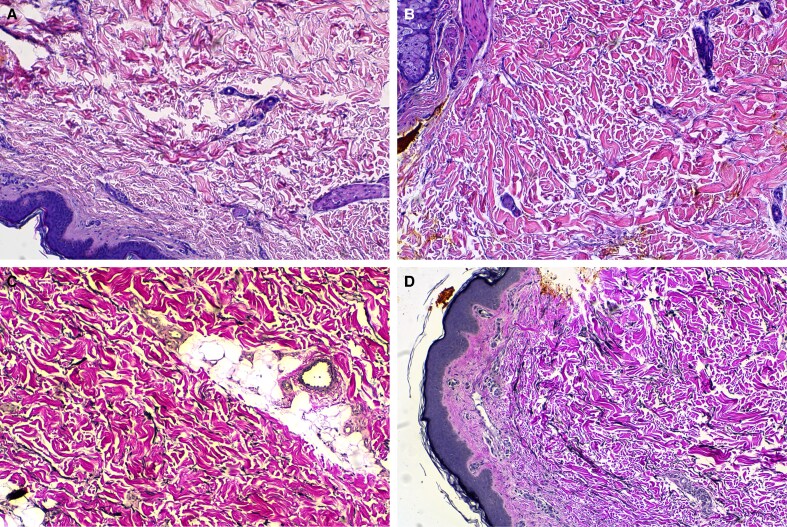
Example image of a collagen histology slide (A) at screening/baseline with a score of 2 and (B) following targeted verifiable subcision (TVS) and helium plasma radiofrequency (RF) treatment at Day 180 with a score of 3. Example image of an elastin histology slide (C) at screening/baseline with a score of 0 and (D) following TVS and helium plasma radiofrequency treatment at Day 90 with a score of 2. Patient: a 42-year-old, Caucasian. Compared with baseline (A, C), the posttreatment slides (B, D) show more robust collagen bundling and increased elastin content, suggestive of enhanced skin elasticity and structural support following treatment.

Elastin content also increased over time. At baseline, 1 patient had an elastin score of 0, and 4 patients had a score of 1. At D90 (*n* = 4), elastin scores increased to 1 in 2 patients and to 2 in 2 patients. By D180 (*n* = 4), elastin scores ranged from 1 to 3, with 2 patients scoring 2, 1 patient scoring 3, and 1 patient scoring 1. For representative examples of before and after collagen and elastin histology slides, see Figures 3A-D and 4A-D.

PAS staining demonstrated increased extracellular matrix density and organization, along with visible epidermal thickening ranging from 50 to 100 microns at follow-up. Localized vascular changes and mild inflammation were noted at D90; however, these findings were limited and not associated with any long-term adverse clinical effects. No evidence of excessive fat necrosis or long-term adverse histological changes was observed.

Epidermal thickness and morphology were also evaluated in the 5 patients who underwent histological sampling. At baseline, epidermal thickness ranged from ∼30 to 80 microns, with several specimens described as having normal architecture and occasional effacement. Immediate posttreatment biopsies demonstrated transient epidermal changes in some samples, including focal vacuolization and minimal associated changes in the underlying fat, consistent with an acute procedural response.

At D90 (*n* = 4), epidermal thickness ranged from ∼40 to 100 microns, with preservation of epidermal architecture and resolution of acute posttreatment changes. By D180 (*n* = 4), epidermal thickness ranged from ∼50 to 100 microns, with overall maintenance or increase in epidermal thickness compared with the baseline. Occasional vacuolization was noted in isolated samples at later time points but was limited in extent and not associated with adverse clinical findings. Collectively, these findings suggest epidermal stability with a trend toward increased thickness over time following combined TVS and helium plasma RF treatment, without evidence of sustained epidermal injury.

**Figure 5.**
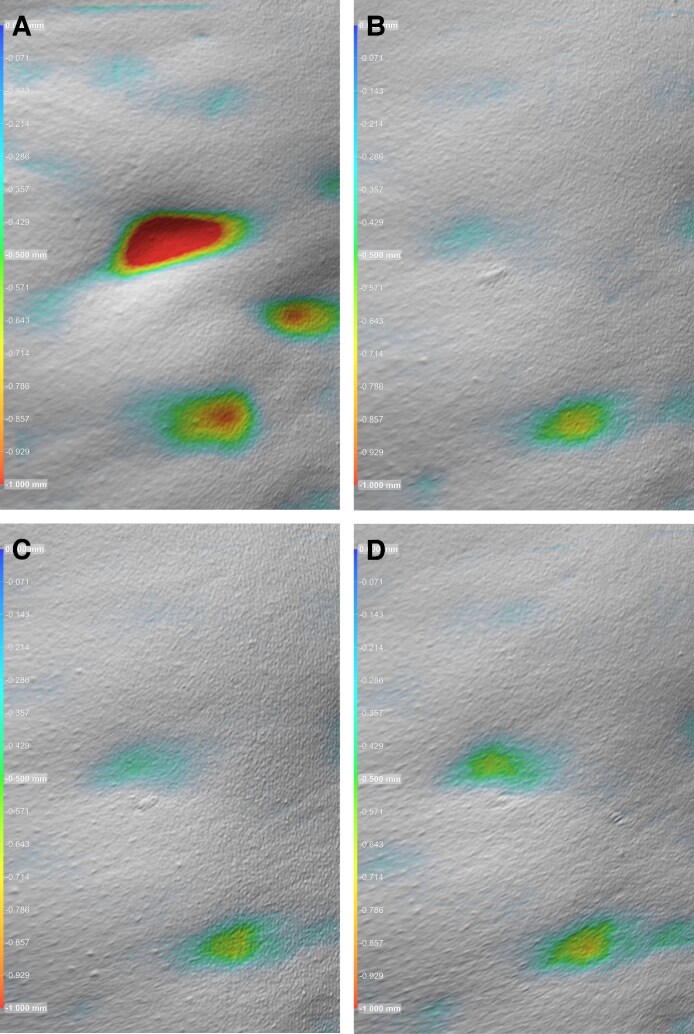
Volumetric image analysis of cellulite depressions (A) at screening/baseline, (B) Day 30, (C) Day 90, and (D) Day 180. Patient: a 45-year-old, Hispanic/Latino.

**Figure 6.**
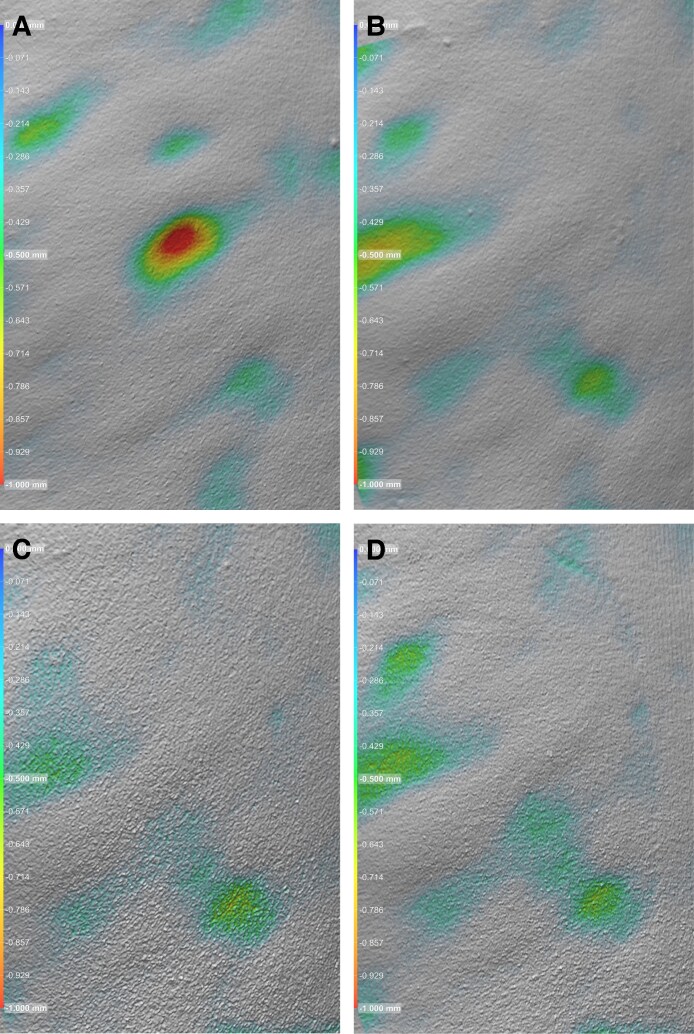
Volumetric image analysis of cellulite depressions (A) at screening/baseline, (B) Day 30, (C) Day 90, and (D) Day 180 (D). Patient: a 45-year-old, Hispanic/Latino.

**Figure 7.**
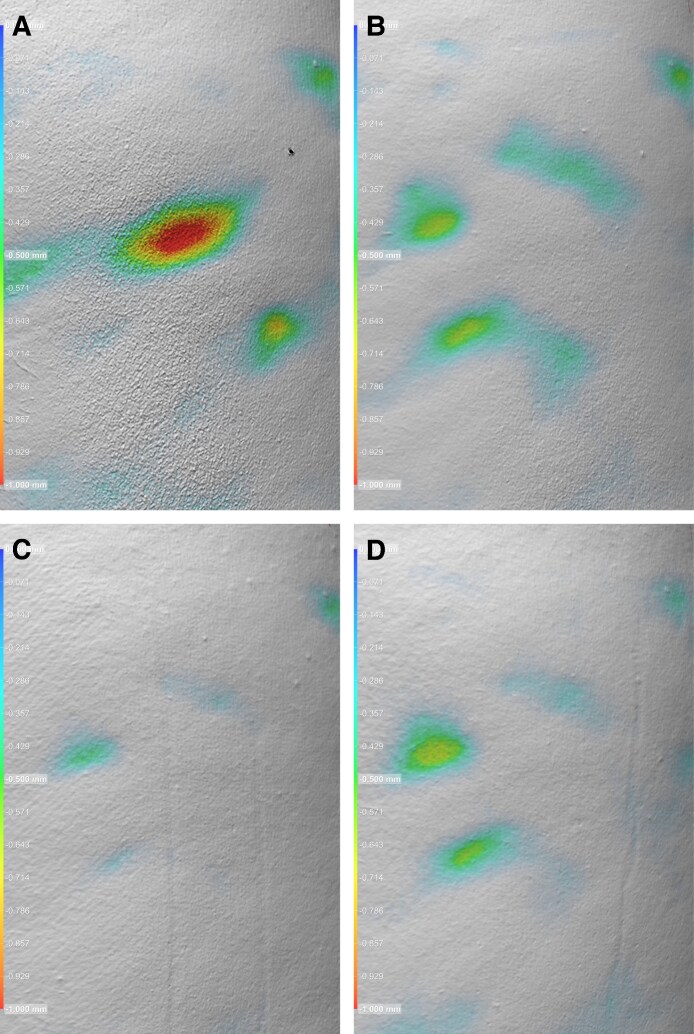
Volumetric image analysis of cellulite depressions (A) at screening/baseline, (B) Day 30, (C) Day 90, and (D) Day 180. Patient: a 45-year-old, Caucasian.

Volumetric image analysis was performed by Canfield Scientific on a subset of patients (*n* = 3) to evaluate changes in cellulite dimple volume (cc), surface area (cm^2^), and depth (mm) from baseline through follow-up. The analysis included both quantitative and visual assessments, with representative results shown in Figures 5A-D, 6A-D, and 7A-D and summarized in Table 3.

**Table 3.** Canfield Scientific Volumetric Image Analysis of Cellulite Depressions

Image	Time point	Volume (cc)	Surface area (cm^2^)	Depth (mm)
Figure 5A	Baseline	0.43	7.01	−2.10
Figure 5B	Day 30	0.40	2.14	−0.35
Figure 5C	Day 90	0.07	2.96	−0.44
Figure 5D	Day 180	0.11	3.79	−0.62
Figure 6A	Baseline	0.28	6.50	−1.21
Figure 6B	Day 30	None detected	None detected	No depth detection contiguous with center
Figure 6C	Day 90	None detected	None detected	No depth detection contiguous with center
Figure 6C	Day 180	None detected	None detected	No depth detection contiguous with center
Figure 7A	Baseline	0.37	7.16	−1.41
Figure 7B	Day 30	None detected	None detected	No depth detection contiguous with center
Figure 7C	Day 90	None detected	None detected	No depth detection contiguous with center
Figure 7D	Day 180	None detected	None detected	No depth detection contiguous with center

In Figure 5, the cellulite depression demonstrated measurable improvement across all metrics. Volume decreased from 0.43 cc at baseline to 0.40 cc at D30, 0.07 cc at D90, and 0.11 cc at D180. Surface area improved from 7.01 to 2.14, 2.96, and 3.79 cm^2^, respectively. Dimple depth was reduced from −2.10 mm at baseline to −0.35 mm at D30, −0.44 mm at D90, and −0.62 mm at D180.

Figure 6 shows complete resolution of the central depression following treatment. Baseline values were 0.28 cc in volume, 6.50 cm^2^ in surface area, and −1.21 mm in depth. At D30, D90, and D180, no measurable volume, surface area, or depth was detected contiguous with the dimple center.

A similar response was observed in Figure 7, where baseline measurements were 0.37 cc, 7.16 cm^2^, and −1.41 mm. All follow-up assessments through D180 showed no detectable dimple volume, surface area, or central depth, indicating sustained correction of the depression.

The treatment was well tolerated, with minimal adverse effects with 1 patient prescribed Tylenol #3 for mild discomfort at D7. Two patients (9%) experienced mild adverse events that were deemed likely related to the procedure (Table 4). One case of hematoma was reported, resolving within 20 days following the drainage of 14 cc of fluid. Another patient experienced transient shortness of breath, attributed to helium exposure, which resolved without intervention in 4 days. No serious adverse events were observed throughout the study, and no patients discontinued participation because of treatment-related complications.

**Table 4.** Summary of Study Population and Safety Outcomes

Patient ID	Age	BMI	Ethnicity	Treatment area	Adverse event	Severity	Management	Resolution time
106	43	21.4	Caucasian	Thighs	Hematoma	Mild	Drainage (14 cc)	Day 20
108	29	26.6	Hispanic/Latino	Thighs	Transient dyspnea (likely helium related)	Mild	None	Day 4

## DISCUSSION

The findings from this investigator-initiated study support the safety and efficacy of combining TVS with helium plasma RF energy for the treatment of cellulite and skin laxity in the thighs and buttocks. Quantitative imaging analysis, qualitative outcomes, and histological evidence consistently demonstrate progressive improvement in tissue structure and aesthetic appearance up to 180 days posttreatment.

Cellulite pathophysiology is multifactorial, involving fibrous septa tethering, dermal atrophy, and hypodermal fat protrusion.^4^ The mechanistic rationale for our approach reflects these complexities: TVS targets the tethering fibroseptal bands, whereas helium plasma RF provides controlled subdermal heating, which promotes collagen and elastin remodeling and dermal thickening. Histological findings in this study confirmed increased collagen and elastin content, as well as extracellular matrix integrity over time, aligning with established timelines for thermal-induced tissue remodeling. In addition, PAS staining revealed increased extracellular matrix density and organization, supporting the hypothesis that helium plasma RF contributes to dermal restructuring. These findings suggest that the combined treatment promotes progressive dermal regeneration through both fibroseptal release and biostimulatory remodeling.

Our findings build on earlier work with 1440 nm Nd:YAG laser technologies, which demonstrated that thermal subcision could both release fibrous septa and remodel dermal structures. DiBernardo (2011) reported durable improvements and neocollagenesis at 1-year posttreatment, whereas DiBernardo et al (2013) validated this approach in a multicenter study with a 3-step laser protocol.^7,8^ Katz further demonstrated measurable increases in skin thickness and elasticity through 12 months, reinforcing the value of energy-based remodeling in cellulite treatment.^9^ These studies highlight a foundational rationale for combining mechanical and thermal modalities, an approach we further advance using helium plasma RF.

Our findings are consistent with broader literature on the role of energy-based devices to address skin laxity and mechanical release of fibroseptal bands to address cellulite. A comprehensive review by Gabriel et al highlighted that combination strategies, particularly those incorporating energy-based technologies, yield superior outcomes compared with monotherapy approaches, by targeting both fibroseptal structures and dermal quality.^12^

The safety and efficacy profile observed in our study also echoes findings from a multicenter trial conducted by Ruff et al, which evaluated helium plasma RF in the neck and submental region. In that study, the authors demonstrated 82.5% clinical effectiveness, with clinical data supporting subdermal tissue contraction.^13^ These same thermal effects contribute to improvements in skin laxity and contour in other body regions, such as the thighs and buttocks, as seen in our study.

Additionally, in a recent multicenter pivotal study, Stevens et al demonstrated the safety and efficacy of a single-session, TVS procedure for cellulite. At 12 months, 93.9% of patients showed improvement on the GAIS score, with 73.8% of patients achieving a ≥1-point reduction on the cellulite severity scale.^11^ These results underscore the importance of directly releasing fibrous septa to address the structural component of cellulite. Longer-term data from Kaminer et al also showed durable results extending out to 5 years using tissue-stabilized guided subcision, further validating the long-term benefit of TVS.^10,14^ Our approach builds on these findings by incorporating helium plasma RF to also stimulate collagen remodeling, thereby addressing both the architectural and textural components of cellulite.

The percentage of patients demonstrating improvement increased from 38% at D30 to 68% at D90 and D180, indicating progressive collagen remodeling and long-term treatment efficacy.

These findings suggest that physicians observed continued improvements beyond the initial treatment period, aligning with the objective quantitative imaging findings and histological evidence of ongoing collagen and elastin regeneration.

Although PGAIS and SGAIS improvement rates in this study (68% and 45% at D180, respectively) may appear modest compared with cellulite-focused studies demonstrating superior outcomes, these results should be interpreted considering the study's broader therapeutic objectives.^11^ Notably, the SGAIS measures perceived visual improvement compared with baseline photography but does not capture overall patient satisfaction. This distinction is important, as perceived improvement may not fully reflect how satisfied patients feel about the treatment outcome. Patient satisfaction was not separately evaluated, representing a limitation in this study.

The early peak in perceived improvement may reflect temporary postprocedural effects, such as swelling, which diminished by later time points. Despite the decline in patient-reported improvement at later time points, physician assessments and histological analysis demonstrated sustained remodeling, indicating that objective tissue improvements persisted even when not readily perceived by patients. This suggests a possible disconnect between subjective perceptions and objective outcomes, reinforcing the need for comprehensive evaluation tools that include both clinical observation and patient-reported satisfaction metrics in future studies. Furthermore, unlike approaches that target isolated cellulite dimples, this investigation addressed multifactorial conditions, fibrous septa, dermal laxity, and adipose protrusion, using a dual-modality treatment. The protocol limited treatment to a small number of cellulite dimples per patient (maximum 4 per thigh), and patients likely evaluated outcomes based on the appearance of the entire thigh rather than focal dimple correction. As a result, perceived improvements may have been diluted. These more modest scores likely reflect the greater complexity of the condition being treated and the comprehensive, rather than focal, approach employed.

Contour irregularities are a recognized risk when subcision is performed aggressively or in areas with significant preexisting laxity. Based on anecdotal clinical experience, it may occur when treatment extends beyond marked areas, when excessive or overlapping markings are made, or when regions with severe laxity are marked and treated. These risks can be minimized through careful patient selection and adherence to marking protocols, as demonstrated in this study, which limited treatment to discrete dimples in patients with mild-to-moderate cellulite.

Although the role of helium plasma RF in preventing postsubcision irregularities has not been fully characterized, the progressive extracellular matrix remodeling observed histologically, along with increased elastin and collagen content and epidermal thickening, suggests that dermal reinforcement may play a protective role in preserving contour and skin integrity. Future studies are warranted to determine whether this biostimulatory effect reduces the risk of adverse surface irregularities in higher-risk patient populations. This study is limited by its small sample size and relatively short follow-up period. Additionally, the PGAIS was completed by the treating investigator, which may introduce bias. Future studies with larger cohorts, side-by-side evaluations with alternative cellulite and skin laxity treatment methods, and extended follow-up are warranted to validate and expand upon these findings.

These findings validate the combination approach as a minimally invasive, effective, and safe treatment for cellulite and skin laxity. Building on these findings, this study suggests that combining TVS and helium plasma RF offers a safe, minimally invasive, and effective approach for both the structural and aesthetic manifestations of cellulite and skin laxity. These results contribute to the growing body of evidence supporting the role of combination therapies utilizing TVS and helium plasma RF in aesthetic dermatology and justify further investigation into its long-term outcomes and broader applications.

## CONCLUSIONS

This preliminary study demonstrated that combining TVS with helium plasma RF was associated with improvements in the appearance of cellulite and skin laxity through 180 days posttreatment. Quantitative imaging, qualitative assessments, and histological analysis supported progressive tissue remodeling and extracellular matrix enhancement. Although these findings suggest potential benefits of this dual-modality approach, the small cohort and short follow-up period limit definitive conclusions. Further studies with larger populations and extended follow-up are needed to confirm these initial observations and optimize treatment protocols.
